# Determination of red blood cell deformability using centrifugal force in a three-dimensional-printed mini-disk (3D-PMD)

**DOI:** 10.1371/journal.pone.0197619

**Published:** 2018-05-22

**Authors:** Hyunjung Lim, Seung Min Back, Jeonghun Nam, Hyuk Choi

**Affiliations:** 1 Department of Medical Sciences, Graduate School of Medicine, Korea University, Guro-gu, Seoul, Korea; 2 Department of Laboratory Medicine, College of Medicine, Korea University, Guro-gu, Seoul, Korea; 3 Department of Emergency Medicine, College of Medicine, Korea University, Guro-gu, Seoul, Korea; The Ohio State University, UNITED STATES

## Abstract

Measuring red blood cell (RBC) deformability has become important for clinical disease diagnostics. Various methods for measuring RBC deformability have been developed; however, they require costly and large instruments, long measuring time, and skilled personnel. In this study, we present a three-dimensional-printed mini-disk (3D-PMD) for measuring RBC deformability to overcome the previous limitations. For a miniaturized and low-cost setup, the 3D-PMD was fabricated by a 3D printing technique, which had not yet been used for fabricating a lab-on-a-compact disk (LOCD). Using a 3D printing technique, a multi-layered fluidic channel on the mini CD could be fabricated easily. During rotation by a spinning motor, the difference of the length of compressed RBCs in the fluidic channel was measured and analysed as compressibility indices (CIs) of normal and glutaraldehyde-treated hardened RBCs. The rotation speed and time were decided as 3000 rpm and 30 min, respectively, at which the difference of CI values between normal and hardened RBCs was largest (CI_normal_-CI_hardened_ = 0.195).

## Introduction

Rheological properties of red blood cells (RBCs) play critical roles in blood flow, especially in microcirculation. RBCs must change their shape to pass through capillaries in the microcirculation network, which have smaller diameters (3–7 μm) than RBCs themselves (7.8 μm on average). In addition, RBC deformability significantly affects blood viscosity. Reduced RBC deformability can increase blood viscosity and flow resistance, which can cause pathophysiologically high blood pressure to maintain blood flow [1,2]. Changes in RBC deformability can be induced by several pathophysiological conditions, including malaria, diabetes, sickle cell anaemia, obesity, bacterial inflammation, and blood storage [3–11].

To measure RBC deformability, various standard methods have been used, such as micropipette aspiration, atomic force microscopy (AFM), and the optical tweezer [12–15]. However, despite single cell-based precise measurement results, these standard tools are time-consuming, laborious, and complicated. To address the limitations, other techniques have been developed, which can achieve rapid measurement and simple operation [16–19]. Among them, optical measurement using laser diffraction has become the primary method due to its convenience and sensitivity [17]. In diluted RBC suspensions, multiple RBCs are deformed under various shear conditions [18,19] and the diffraction pattern of deformed RBCs is observed to evaluate the average RBC deformability. The optical measurement technique still has been limited, since it requires large volume of blood sample and a chamber cleaning process after every measurement. In addition, using these instruments, simultaneous multi-processing cannot be achieved to enhance the throughput.

Due to the recent development of microfluidic techniques, various microfluidic approaches have been proposed to measure RBC deformability under physiological shear flow conditions in a microfluidic channel by channel geometries and flow characteristics [20–27] to induce RBC deformation. However, lithographic processes in cleanroom facilities are required to fabricate the microfluidic device. Also, for observation, expensive optical measurement systems such as high speed camera and high intensity light source are required and the number of RBCs which can be recorded is limited due to the limited memory capacity of the high speed cameras.

Recently, lab-on-a-compact disk (LOCD) methods have gained much attention, due to their notable advantages including cost-effective device fabrication, simple flow control by centrifugal force, compact instrumentation, and multi-processing on a single CD [28,29]. Therefore, LOCD platforms have been used for a variety of applications, such as valving [30], metering [31], mixing [32,33], and separating [34,35]. The LOCD technique has been applied to blood analysis to determine haematocrit and blood cell separation [36–38]. More recently, three-dimensional (3D) printing has been widely used due to its advantages such as low cost, easy access, various materials including biomaterials, and less restriction on design [39,40]. Especially, 3D multi-layered microfluidic structures can be easily fabricated by 3D printing, compared to conventional soft lithography, which is limited by complex, multiple fabrication steps and precise alignment [41]. To the best of the authors’ knowledge, 3D printing has not yet been applied to fabricate a LOCD device. In this study, we developed a 3D-PMD that can measure RBC deformability. By virtue of the advantages of 3D printing, a 3D-structured mini CD was fabricated, containing simple straight channels with two layers. RBC deformability was evaluated by measuring the optical contrast on the volume fraction of cells compressed by centrifugal force during rotation of the 3D-PMD without the use of high speed camera system. This method is not for single cell-based precise measurement but inexpensive, simple, lithography-free and high-throughput compared to previous RBC deformability measurement techniques.

## Materials and methods

### Experimental setup

**Fig 1:**
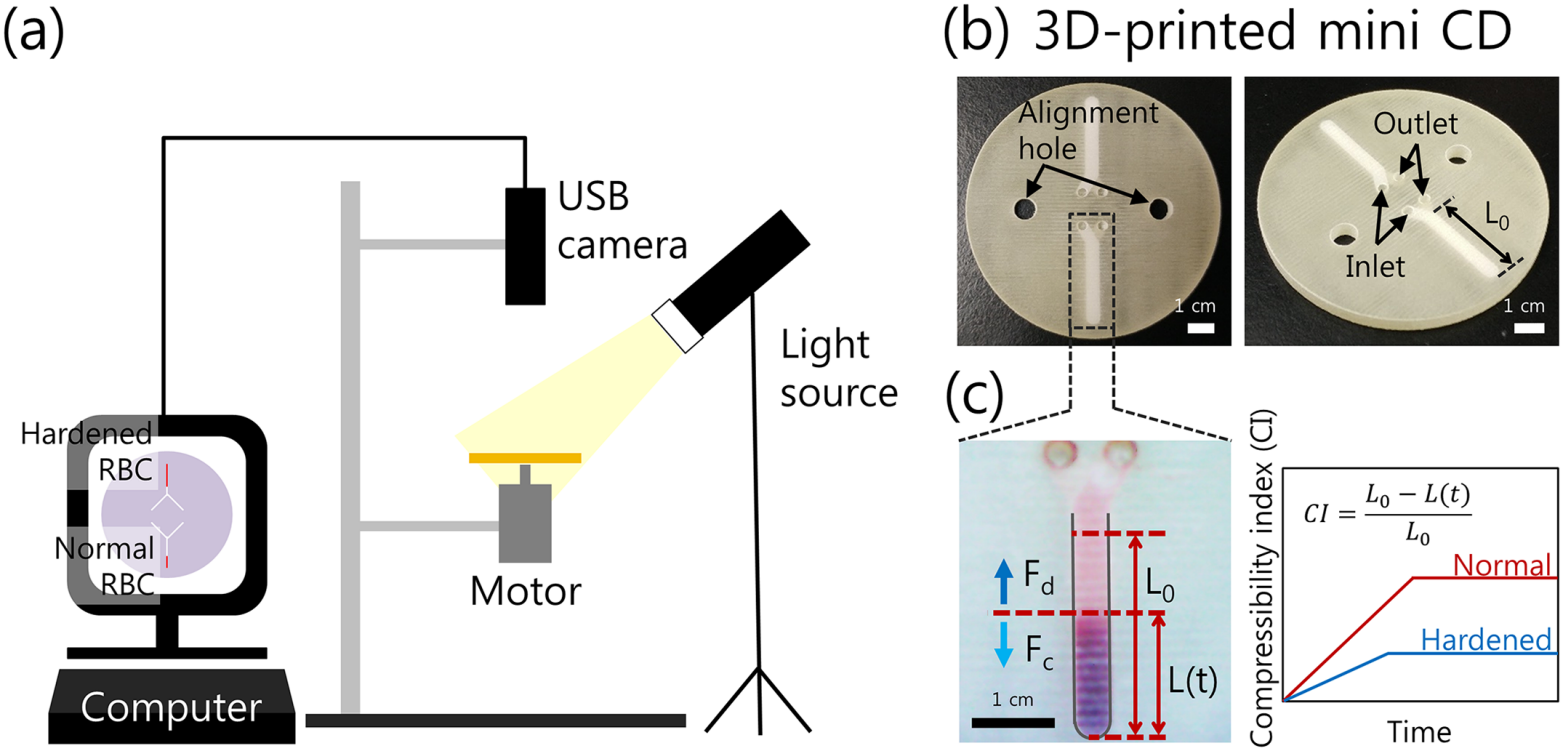
Experimental Setup and working principle. (a) Schematic of the 3D-PMD-based RBC deformability measurement system. (b) Photographs of the 3D-PMD device for measuring RBC deformability. The system contains two channels and two alignment holes that are at opposite sides of the device. The two channels are filled with a normal RBC sample and a hardened RBC sample, respectively. (c) A single unit of a straight channel with one inlet and one outlet and the measurement method of the compressibility index (CI). *F*_*d*_ is the drag force and *F*_*c*_ is the centrifugal force.

Fig 1(a) shows a schematic of the 3D-PMD-based RBC deformability measurement system. It includes the mini CD rotating control system and the optical measurement system. The 3D-PMD device was mounted onto a spinning motor. To rotate the 3D-PMD device, the spinning motor (MDN-4RA3ETA, Minebea-Matsushita Motor Corp., Japan) was used, which was powered by a power supply (DP30-03TP, Toyotech). During rotation, the movement of the cell-liquid interface was recorded using a USB digital microscope (S04-600X) connected to a computer, with a strobe light. To acquire the experimental results at the fixed position, the USB microscope and the spinning motor were aligned using an experimental stand and clamps.

### Device fabrication

The 3D-PMD with a 9-cm diameter was fabricated, which consisted of 2 layers of fluidic channels with 3 layers of connecting parts using stereolithography by a 3D printer (ProJet 3510 HD, 3D systems). VisiJet Crystal (3D systems) was selected as the resin. Details of the multi-layered 3D-PMD device can be seen in Supporting information (S1 Fig). Blood samples can be filled into the fluid channel by pipetting while avoiding air bubble formation. The width and height of the fluidic channel in the 3D-PMD device were 2 mm and 300 μm, respectively. Two fluidic channels were filled with normal RBC sample and hardened RBC sample, respectively, during the measurement. The middle layer contains the holes that connect the top and bottom layers of the fluidic channels. Every layer includes two holes at the opposite sides of the device to align the device layers. Fig 1(b) shows photographs of the integrated 3D-PMD device for measuring RBC deformability.

### Reagent and sample preparation

Human whole blood samples were obtained from the Korean Red Cross complied with safety regulations through an Institutional Review Board (IRB) approved collection method (KUGH17177-001, approved by Korea University Guro Hospital). The need for consent was waived by the ethics committee. Blood samples were drawn, mixed with an anticoagulant and stored at 4°C. All experiments were performed within one hour after treatment. RBCs were obtained by centrifuging whole blood at 800×*g* for 10 min. After centrifugation, plasma and other cells (white blood cells and platelets) in the buffy coat were removed. RBCs were washed three times with the Phosphate Buffered Saline (PBS) to avoid plasma-protein induced aggregation effect and resuspended in PBS at a known haematocrit of 40%. To rigidify RBCs, normal RBCs were exposed to 0.01 and 0.02% glutaraldehyde (GA, Sigma, St. Louis, MO, USA) at 25°C for 30 min [42]. GA has been commonly used for RBC deformability studies to rigidify RBCs by cross-linking the cytoskeletal proteins [20]. Following the treatment, hardened RBCs were washed three times with PBS and resuspended in PBS to 40% haematocrit.

### Working principle

Fig 1(c) shows a single unit of a fluidic channel in the 3D-PMD device. Initially, the fluidic channel is filled with blood suspension to a length *L*_*0*_. During rotation, the centrifugal force (*F*_*c*_) overcomes the drag force (*F*_*d*_) at a specific rotational speed, and RBCs start to precipitate in the radial direction. Therefore, RBCs are packed at the end of the fluidic channel to the length *L(t)* at time *t* after the onset of rotation. Compressibility index (CI) can be defined as below.

$$CI\;=\;\frac{L_{0}-L(t)}{L_{0}}$$ (1)

*L*_*0*_ is the initial length of the blood sample in the fluidic channel before rotating the 3D-PMD device, which is the same as the length of the fluidic channel. According to its definition, CI will have a value between 0 and 1. During the RBC sedimentation process, normal, deformable RBCs are more compressed and densely packed compared to hardened RBCs, so normal RBC samples have higher CI values compared to those of hardened RBC samples under the same rotational conditions. During rotation of the 3D-PMD device, time-dependent *L(t)* values are measured to determine the rotational condition (rotational speed and time) with the largest differences between normal and hardened RBCs.

## Results and discussion

**Fig 2:**
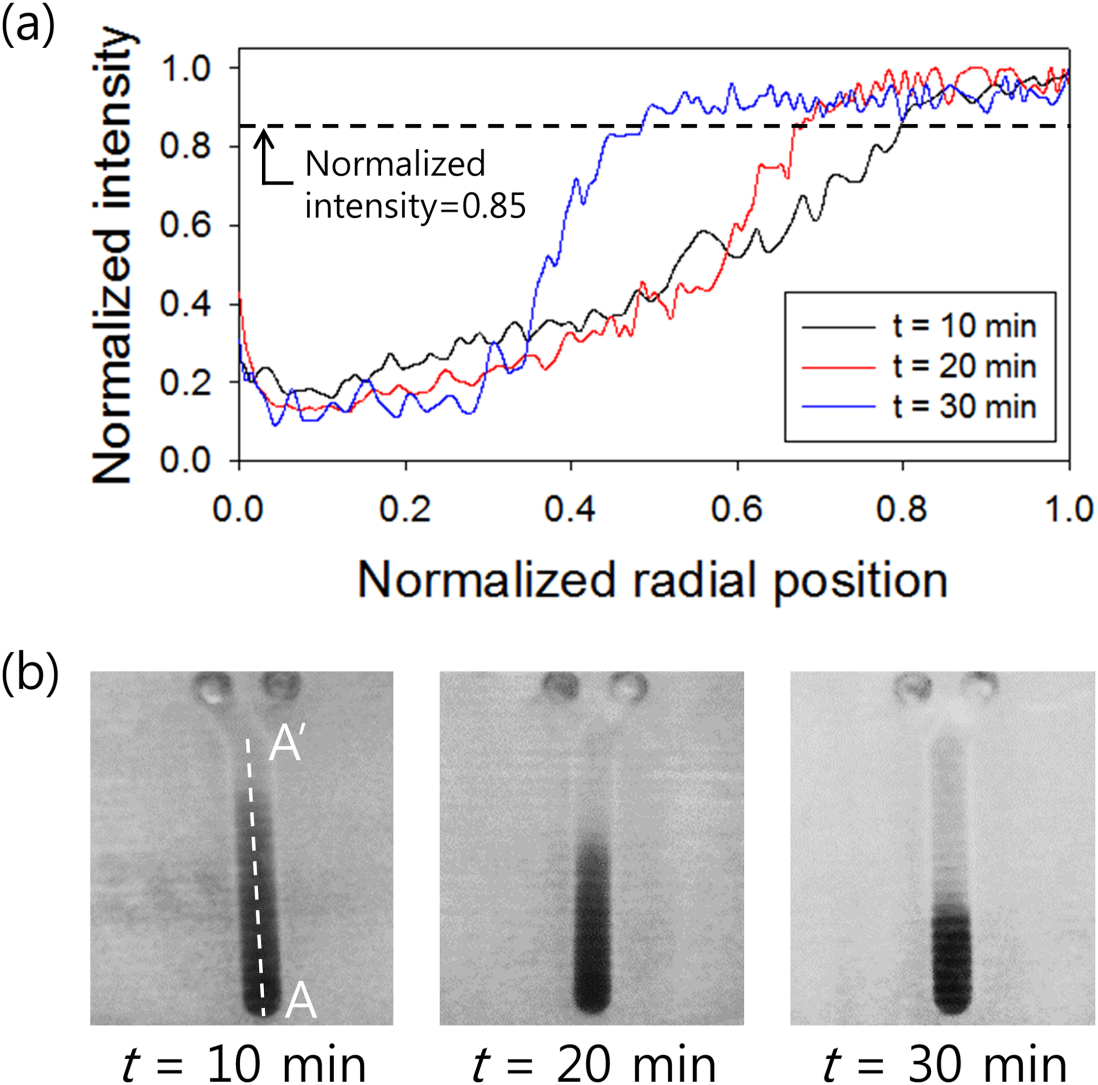
Analysis of CI using normalized intensity. (a) Normalized intensities in the fluidic channel of the 3D-PMD device as a function of normalized radial position at different time rotation durations (10, 20, and 30 min). The dotted line indicates the normalized intensity of 0.85. (b) Photographs of a single unit of the fluidic channel 10, 20, and 30 mins after beginning 3D-PMD rotation. White dotted line (A-A’) shows the radial position in the fluidic channel for intensity analysis.

To determine packed cell length *L(t)* in the fluidic channel for CI analysis, intensities in the fluidic channel were analysed. The 3D-PMD device, which was filled with normal RBC sample was rotated at fixed rotational speed of 3000 rpm. Photographs of a single unit of the fluidic channel which were taken after 10, 20, and 30 minutes of 3D-PMD rotation, were used for analysis. Images were converted to grayscale with 256 levels between 0 (black) and 255 (white) from RGB and the intensity profiles were analysed using Image J. Normalized radial position indicated the distance from the end of the fluidic channel along the white dotted line (A-A’) as seen in Fig 2(b), which was normalized to the total length of the channel. Therefore, the normalized radial position values of the end of the fluidic channel and *L*_*0*_ were 0 and 1, respectively. Measured intensity was normalized with the maximum intensity value from each case; therefore, the normalized intensities at the dark region with cells and the bright region without cells were 0 and 1, respectively. The critical value of the normalized intensity was chosen as 0.85 at which the slope started to decrease due to the saturation of the normalized intensity. As shown in Fig 2, as the rotation duration became longer, the intensities in the fluidic channel became closer to 1 due to RBC sedimentation.

In Fig 2(a), *L(t)* can be determined as the normalized radial position at which the normalized intensity has a value lower than 0.85. For example, at *t* = 10 min in Fig 2(b), the normalized intensity became 0.85 at *L(t = 10)* = 0.8, so that the CI_t = 10_ would be calculated as 0.2. Likewise, the CI values for *t* = 20 and *t* = 30 were approximately 0.35 and 0.54, respectively.

**Fig 3:**
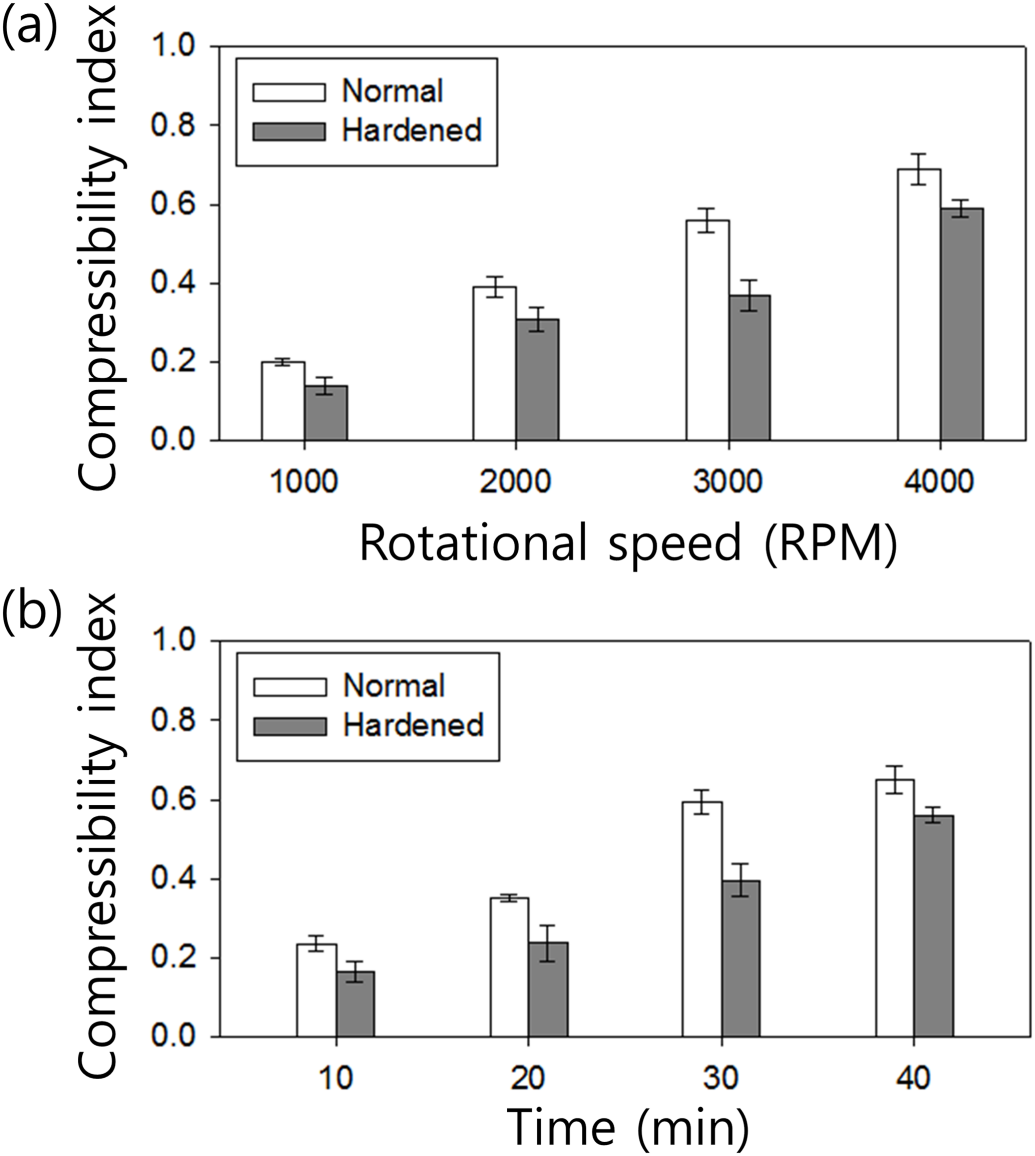
Determination of the rotational conditions for measuring RBC deformability. Compressibility index of normal and hardened RBCs (a) at various rotational speeds after 25 min and (b) depending on various rotation times with a fixed rotational speed at of 3000 rpm. The standard deviation shows the measured values from ten different measurements (n = 10).

To determine the rotational conditions for measuring RBC deformability, CI values of normal and hardened RBCs were evaluated depending on the rotation time and rotational speed. The rotation time shorter than 10 min and the rotation speed lower than 1000 rpm were not suitable to discriminate the CI values of normal and hardened RBCs (data not shown). Fig 3(a) shows the CIs as a function of the rotational speed at the fixed rotation time of 25 min. The voltage conditions applied to the spinning motor was determined to achieve the rotational speed of 3D-PMD between 1000 and 4000 rpm; see the voltage-dependent rotational speed included in the supporting information section for additional details (S2 Fig). Depending on the rotational speed, the final fraction of RBCs in the total length of the fluidic channel changes. Even at low rotational speeds, normal RBCs can be more packed at the end of the fluidic channel compared to hardened RBCs, due to their deformability difference. Therefore, at the rotational speed of 1000 rpm, CIs of normal RBC samples were higher (CI_normal_ = 0.2±0.01) than those of hardened RBC samples (CI_hardened_ = 0.14±0.02). As rotational speed increased, RBCs compressed more densely, so that *L(t)* became shorter. The differences between CIs of normal and hardened RBCs were 0.19 and 0.1 for rotational speeds of 3000 and 4000 rpm, respectively. At 4000 rpm, due to high centrifugal force, hardened RBCs were more densely packed and CI_4000rpm_ of hardened RBCs became similar to that of normal RBC samples. Therefore, the optimal rotational speed was 3000 rpm for measuring deformability.

Fig 3(b) shows the CIs as a function of the rotation time at the fixed rotational speed of 3000 rpm, as determined from data in Fig 3(a). Over the range of rotation time from 10 to 40 min, normal RBC samples exhibited higher CI values compared to those of hardened RBC samples, due to RBC deformability. For normal RBCs, CI increases rapidly with increasing rotation time until *t* = 30 min. As the rotation time increased to *t* = 40 min, the increased rate of CI declined. At 30 ≤ *t* ≤ 40 min, sunken RBCs filled in the empty space between RBCs and packed more densely at the end of the fluidic channel due to the centrifugal force, therefore *L(t = 40)* decreased slowly. On the other hand, since hardened RBCs tend not to compress, their CI increase rate did not change much depending on rotation time. As shown in Fig 3(b), the difference in CIs between normal and hardened RBCs increased and reached a maximum value at *t* = 30 min. Then, it decreased again upon further rotation until t = 40 min. Therefore, the optimal rotation time for sensitively measuring RBC deformability was determined to be 30 min.

**Fig 4:**
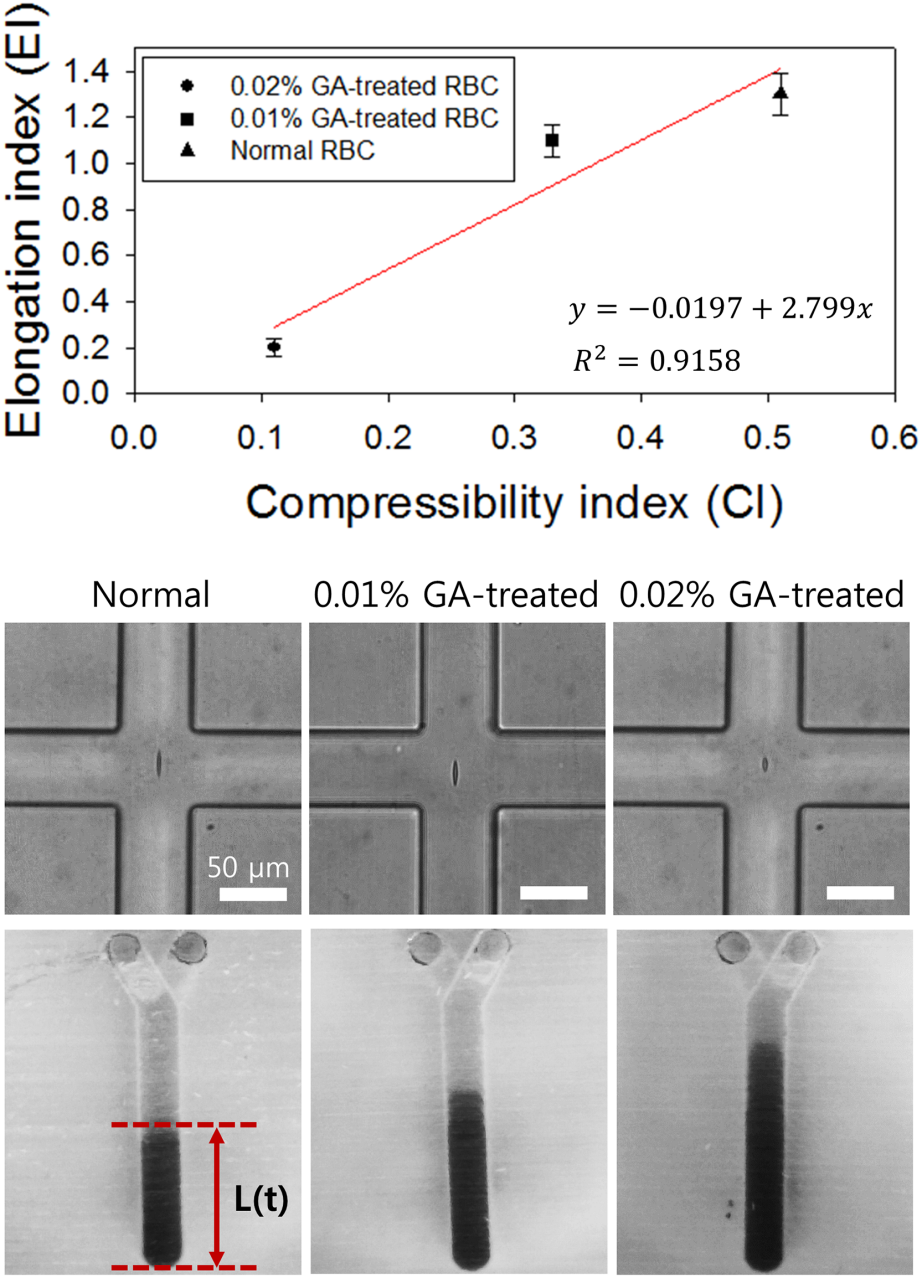
Validation of CIs measured using a 3D-PMD device. Relation between compressibility indices (CIs) measured using a 3D-PMD device and elongation indices (EIs) measured using a viscoelastic cell deformability measurement technique depending on RBC rigidity. The standard deviation shows the measured values from ten different measurements (n = 10). Photographs of stretched RBCs in a cross-shaped channel and single units of the fluidic channel of the 3D-PMD device.

To validate the 3D-PMD device for RBC deformability measurement, we compared deformabilities of normal RBCs and hardened RBCs measured using our 3D-PMD device and the viscoelastic cell stretching measurement method, as shown in Fig 4. The details of the viscoelastic cell deformability measurement technique were previously described [25]. Elongation index (EI) can be defined as below.

$$EI\equiv \frac{L-\overset{-}{L_{0}}}{\overset{-}{L_{0}}}$$ (2)

*L* is the maximum length of a deformed RBC and $\overset{-}{L_{0}}$ is the average length of non-deformed RBCs. To mimic less-deformable RBCs, normal RBCs were chemically treated with 0.01 and 0.02% glutaraldehyde. Comparing the results (EIs and CIs), the correlation coefficient *R*^*2*^ from the Pearson’s correlation test was 0.9158 (*y* = -0.0197+2.799*x*). This shows that RBC deformability measurement acquired by the two techniques were very strongly correlated.

From time-dependent packed cell lengths measured during 3D-PMD device rotation, altered RBC deformability could be measured. Various physiological phenomena, such as diabetes mellitus, malaria infection, sickle cell disease, stored blood, and RBC aging, are generally known to be associated with reduced RBC deformability [10], which requires to be considered to optimize the 3D-PMD device for clinical use. With further optimization including configurations of the 3D-PMD device, the rotational conditions, the optical measurement system, and the surface roughness characterization [43], this 3D-PMD-based RBC deformability measurement system can have the potential to become a useful tool with a thermometer-styled reading not only for clinical diagnosis but also in resource-limited developing countries [44,45].

## Conclusion

A 3D-PMD based RBC deformability evaluation system was developed, which could measure the difference in length of packed RBCs in a fluidic channel during rotation due to centrifugal force, negating additional fluid pumping system. To evaluate RBC deformability, compressibility index (CI) was newly suggested. The rotational speed and the rotation time of the 3D-PMD device were determined to maximize the difference between CI values of normal and hardened RBCs. The use of a disposable 3D-PMD device enables the simple and low-cost measurement of RBC deformability without using expensive optical measurement systems such as high speed camera, high intensity light source, and so on. Moreover, by placing multiple fluidic channels on a single 3D-PMD device, a large number of blood samples can be analysed simultaneously. Therefore, this 3D-PMD-based RBC deformability measurement system can pave the way for clinical and biological analysis of cell deformability.

## Supporting information

S1 Fig Schematic of multilayered 3D-PMD device. (a) Exploded view of the 3D-PMD device containing five different layers. (b) Final 3D modelling of the 3D-PMD device. (c) Five different layers of the 3D-PMD device.
(DOCX)

S2 Fig Rotational speed of a 3D-PMD device mounted on a spinning motor depending on the applied voltage. (DOCX)
