# Supplementary material for: Determination of red blood cell deformability using centrifugal force in a three-dimensional-printed mini-disk (3D-PMD)
Source: PLoS One. 2018 May 22;13(5):e0197619. doi: 10.1371/journal.pone.0197619 (PMC5963765; doi:10.1371/journal.pone.0197619)
Supplement: S1 Fig — (a) Exploded view of the 3D-PMD device containing five different layers. (b) Final 3D modelling of the 3D-PMD device. (c) Five different layers of the 3D-PMD device. (DOCX) [file pone.0197619.s001.docx]

Supporting information

Determination of red blood cell deformability using centrifugal force in a three-dimensional-printed mini-disk (3D-PMD)

Hyunjung Lim^1¶^, Seung Min Back^1¶^, Jeonghun Nam^2,3*^, and Hyuk Choi^1*^

^1^ Department of Medical Sciences, Graduate School of Medicine, Korea University, 80, Guro-dong, Guro-gu, Seoul, 152-703, Korea.

^2^ Department of Laboratory Medicine, College of Medicine, Korea University, 80, Guro-dong, Guro-gu, Seoul, 152-703, Korea.

^3^ Department of Emergency Medicine, College of Medicine, Korea University, 80, Guro-dong, Guro-gu, Seoul, 152-703, Korea.

^*^Corresponding author:

**Jeonghun Nam, PhD** jhnam77@gmail.com;

**Hyuk Choi, PhD** hyuk76@korea.ac.kr

^¶^ These authors contributed equally to this work.

**Assembly of the 3D-PMD device**

Figure S1 shows the three-dimensional structure and each layer of the 3D-PMD device. As shown in an exploded view in Fig. S1(a), the 3D-PMD device had five different layers, 2 layers of fluidic channels with 3 layers of connecting parts which were fabricated by a 3D printer. Each of the five separate layers contributed uniquely to the device structure (Fig. S1 (c)). Capping layer has two input ports and two output ports. Two fluidic layers have the upper and lower fluidic channels which are connected by the connection port at a ‘Via’ layer. Base layer is the substrate which only has the alignment holes. Every layer has two holes at both sides for alignment and connection. The 3D-modeling of the fabricated 3D-PMD device was shown in Fig. S1 (b).

**
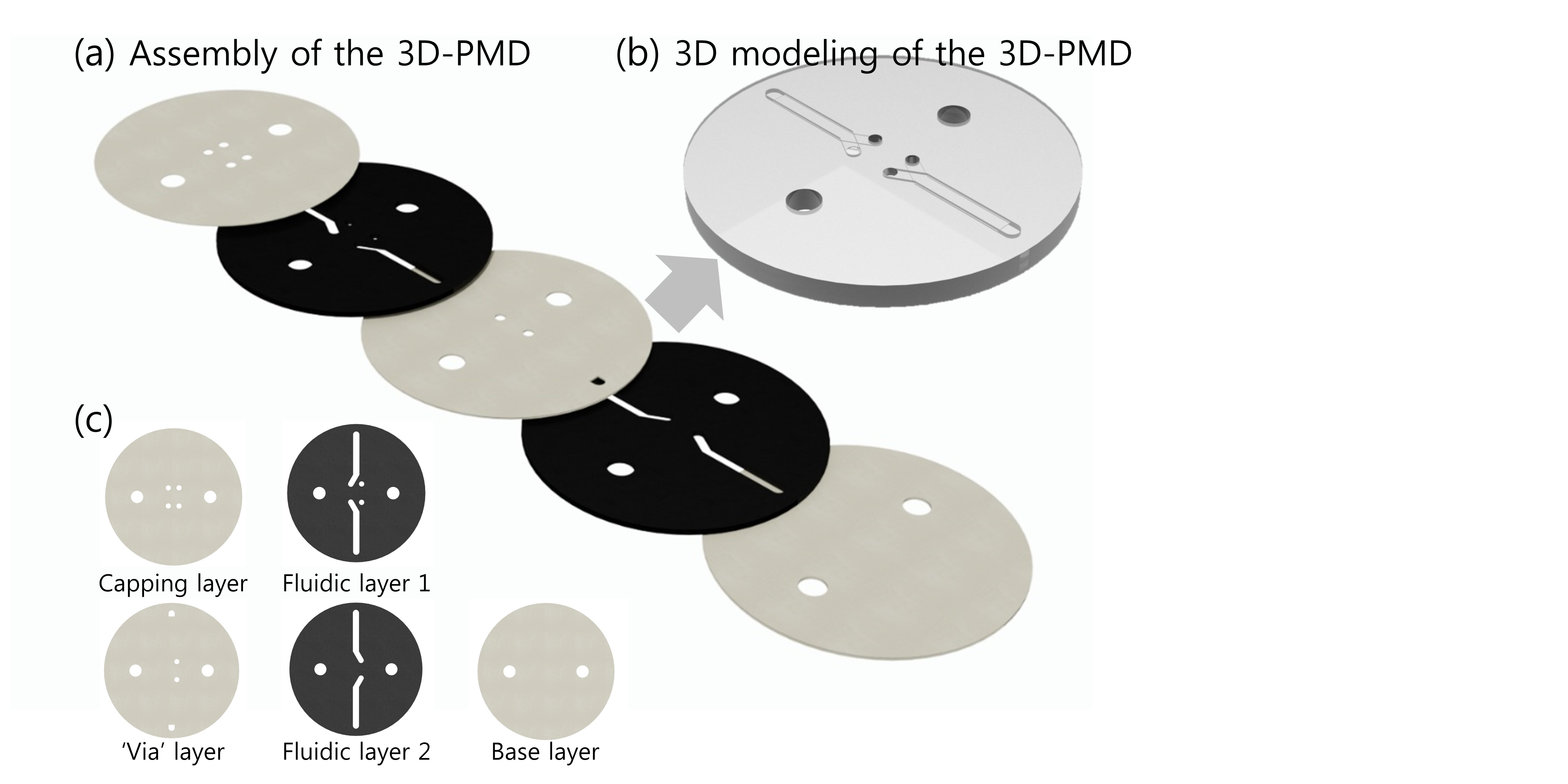
**

**Fig S1.** Schematic of multilayered 3D-PMD device. (a) Exploded view of the 3D-PMD device containing five different layers. (b) Final 3D modelling of the 3D-PMD device. (c) Five different layers of the 3D-PMD device.
