# Supplementary material for: Determination of red blood cell deformability using centrifugal force in a three-dimensional-printed mini-disk (3D-PMD)
Source: PLoS One. 2018 May 22;13(5):e0197619. doi: 10.1371/journal.pone.0197619 (PMC5963765; doi:10.1371/journal.pone.0197619)
Supplement: S2 Fig — (DOCX) [file pone.0197619.s002.docx]

Supporting information

Determination of red blood cell deformability using centrifugal force in a three-dimensional-printed mini-disk (3D-PMD)

Hyunjung Lim^1¶^, Seung Min Back^1¶^, Jeonghun Nam^2,3*^, and Hyuk Choi^1*^

^1^ Department of Medical Sciences, Graduate School of Medicine, Korea University, 80, Guro-dong, Guro-gu, Seoul, 152-703, Korea.

^2^ Department of Laboratory Medicine, College of Medicine, Korea University, 80, Guro-dong, Guro-gu, Seoul, 152-703, Korea.

^3^ Department of Emergency Medicine, College of Medicine, Korea University, 80, Guro-dong, Guro-gu, Seoul, 152-703, Korea.

^*^Corresponding author:

**Jeonghun Nam, PhD** jhnam77@gmail.com;

**Hyuk Choi, PhD** hyuk76@korea.ac.kr

^¶^ These authors contributed equally to this work.

**Measuring the rotational speed of the 3D-PMD device**

To determine the voltage conditions applied to a spinning motor, the rotational speed of the 3D-PMD mounted on the motor was measured by an LED tachometer. The rotational speed showed a linear correlation with the applied voltage (*n*=26.42+987*V*, *n* is the rotational speed and *V* is the applied voltage). To measure RBC deformability, applied voltages ranging from 1−4 V were used.


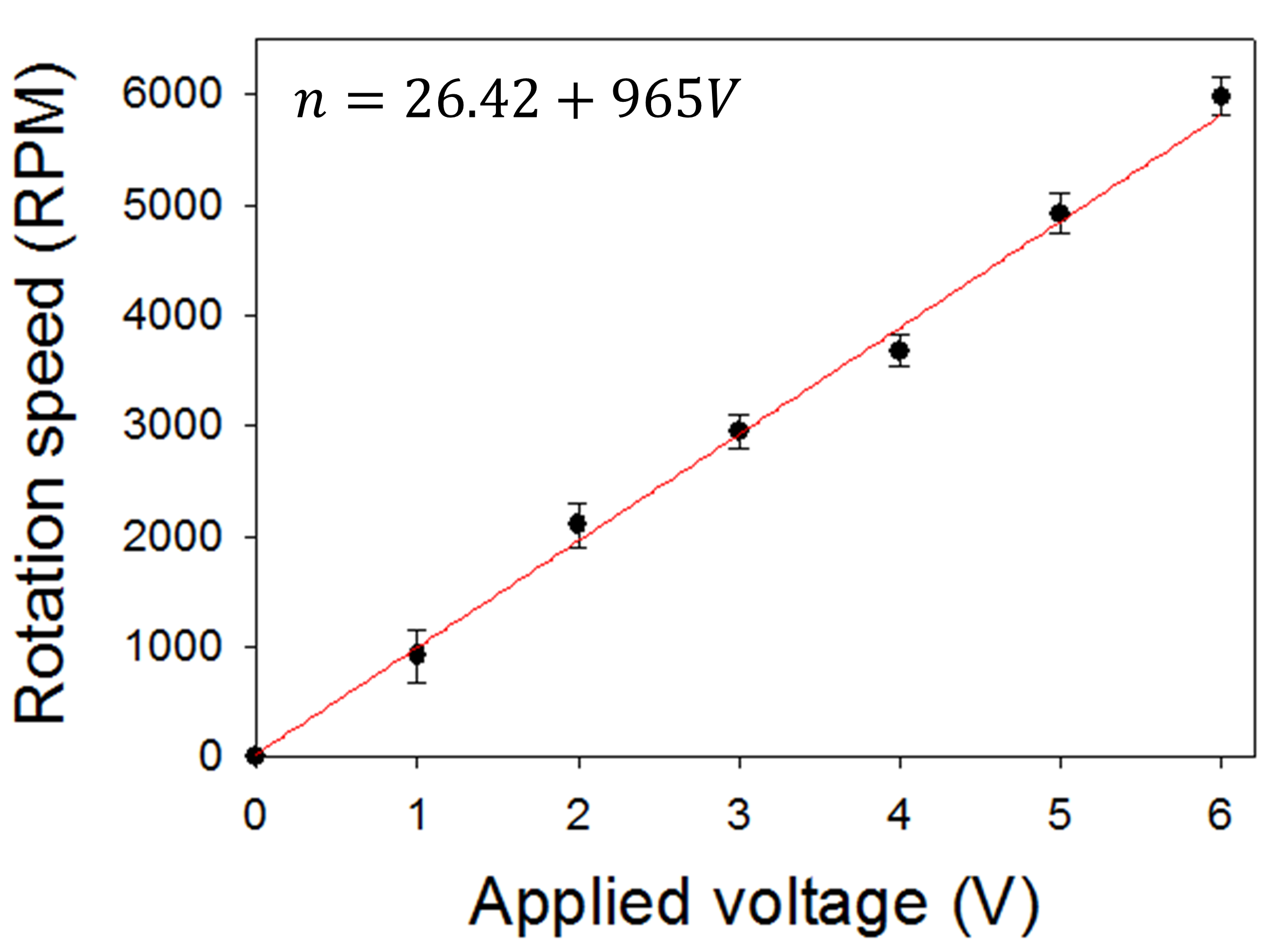


**Fig S2.** Rotational speed of a 3D-PMD device mounted on a spinning motor depending on the applied voltage.
